# Prophylactic anticoagulation in traumatic subdural hematoma

**DOI:** 10.1038/s41598-025-93981-8

**Published:** 2025-03-19

**Authors:** Florian Wilhelmy, Michael Gaier, Gordian Prasse, Börge Bastian, Jürgen Meixensberger, Erdem Güresir, Tim Wende, Johannes Kasper

**Affiliations:** 1 Department of Neurosurgery, University Hospital Leipzig, Liebigstrasse 20, 04103 Leipzig, Germany; 2 Department of Anesthesiology and Intensive Care, University Hospital Leipzig, Liebigstrasse 20, 04103 Leipzig, Germany; 3 Division of Neuroradiology, University Hospital Leipzig, Liebigstrasse 20, 04103 Leipzig, Germany

**Keywords:** Traumatic brain injury, Anticoagulant, Neurosurgery, Perioperative care, Thromboembolism, Postoperative hemorrhage, White matter injury, Neurological manifestations

## Abstract

Severe traumatic brain injury (TBI) with acute subdural hematoma (SDH) is common in neurosurgical care. Especially due to demographic development, it becomes increasingly coincident with preexisting therapeutic anticoagulation and comorbidity, such as atrial fibrillation or coagulative disorders. High-velocity trauma mechanisms become rarer, while low-impact trauma to the skull with acute-on-chronic subdural hemorrhage gets relatively more frequent. In this study we elucidate the timing of perioperative prophylactic AC and its influence on morbidity and mortality as well as complications after neurosurgical treatment. We focused especially on postoperative intracranial hemorrhage (PH) and thromboembolic events (TE). For this retrospective data analysis, 259 patients who suffered from severe TBI with consecutive subdural hematoma between 01/01/2014 and 31/12/2019 were included. We followed up for the length of stay. We assessed various biographical and clinical data for risk factors and focused on the connection between time-point of AC and adverse events. Subgroup analyses were performed for TE and PH that either required surgical intervention or was managed conservatively with radiological follow-up. Statistical analysis was performed using receiver operating characteristic curve analyses, Mann-Whitney U Test, Chi-square test, Fisher’s exact test and univariate binomial logistic regression. P-values below 0.05 were considered statistically significant. TE were relatively rare in this cohort (*n* = 14, 5.4%). The more common adverse event was PH (*n* = 34, 13.1%), with a total of 28 patients (10.8%) needing surgical revision. Though PH was correlated to a delay in AC (*p* = 0.010), there was no correlation between early prophylactic AC and PH (*p* = 0.287) or TE (*p* = 0.444), respectively. Furthermore, only 4 patients had been administered AC before the PH. In this context, AC was delayed purposely after PH, explaining the significant correlation between PH and delayed AC. Occurrence of PH significantly decreased overall survival (*p* = 0.022), while TE did not (*p* = 0.357). Prophylactic AC within 24–48 h after surgery did not result in more frequent PH. Our data on AC timing suggest that PH is not caused by prophylactic AC.

## Introduction and background

During the last decades, the incidence of high-velocity trauma in developed countries has decreased due to safer traffic and demographics^1^. The latter also led to an increase in mild trauma of the elderly, leading to intracranial traumatic bleeding as sequelae of insignificant injuries per se^2^. Severe traumatic brain injuries (TBI) can lead to extensive disability, long-term immobilization and secondary coagulopathy^3–5^, therefore putting trauma patients at risk for coagulative complications such as thromboembolic events (TE) and postoperative intracranial hemorrhage (PH), or even hemorrhage elsewhere then the neurocranium^6–8^.

In the prevention of hypercoagulative disorders, such as pulmonary embolism (PE), deep vein thrombosis (DVT), stroke or myocardial infarction (MI), practitioners regularly use prophylactic anticoagulation (AC) in ICU and perioperative management. It has since been discussed, how AC influences postoperative PH, as it weakens the physiological ability to prevent bleeding^9^. In several studies we have shown the effects of the timing of AC administration, as we struggle to set the pace for AC in guidelines. Mostly, when AC is discussed in surgical context, it focuses on the “IF” instead of “WHEN”^10^. In spontaneous subarachnoid hemorrhage, glioma and meningioma surgery, we could demonstrate that early or continuous prophylactic AC was not correlated to higher bleeding rates in neurosurgical patients^11–13^. In this study, we examined the time-point of AC administration and its influence on common coagulative complications in patients suffering from TBI with SDH.

## Methods

The study was approved by the ethics committee of the Medical Faculty, University of Leipzig (No. 053/19-ek). The need for informed consent was waived by the ethics committee of the medical faculty, Leipzig University, due to the retrospective nature of the study. Data collection and analysis were performed in accordance with the relevant guidelines and regulations.

### Patient selection

We searched our database for all patients undergoing surgery for acute traumatic intracranial bleeding, i.e. acute traumatic subdural hematoma and traumatic intracerebral hemorrhage, at our facility between 2014 and 2019 above the age of 18. Exclusion criteria were isolated extraaxial bleeding, i.e. isolated epidural hematoma, spontaneous ICB with extend to the subdural space without trauma in patient history and subarachnoid hemorrhage (SAH) with extend to subdural space. Acute subdural hematoma without any trauma were excluded. We excluded patients with inconclusive datasets, external operation and minimally invasive procedures such as ICP-monitor placement. Patients who died from complications other than PH or TE before AC could be administered were also excluded.

### Diagnosis of intracranial bleeding and thromboembolism

5 mm and 1.25 mm computed tomography (CT) scans were regularly performed within 6 h after initial event or 24 h after surgery. Patients displaying new neurological deficits or insufficient awakening after surgery were CT-scanned immediately.

**Fig. 1:**
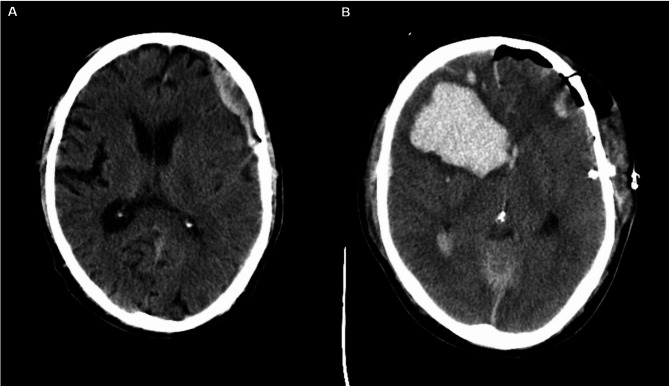
CT scan of traumatic brain injury with subdural hematoma before (A) and after (B) surgical evacuation. Although the good clinical state and equipose situation during surgery allowed for the evacuation via craniotomy, postoperative intracranial hemorrhage occured postoperatively. Notably, no prophylactic anticoagulation had been administered yet.

Postoperative or spontaneous recurrent hemorrhage was defined as radiologically diagnosed intracranial blood volume with mass effect, as independently described by a senior neuroradiologist. As this study is retrospective, we scanned written radiological assessment for the term “re-bleeding” (german: Nachblutung). Postoperative hemorrhage, which was defined without knowledge of this study, was reviewed again during data assessment by the author and a trained neuroradiologist (GP). We included bleedings that have been radiologically identified independently to increase sensitivity for PH. We divided into subgroups for PH in CT diagnostics and PH with the necessity for surgical revision and analyzed accordingly. If not otherwise stated, all CT-morphological PH were included in the analysis, regardless of therapeutic procedure. Figure 1 shows a typical example of PH.

TE were defined as pulmonary artery embolism or deep vein thrombosis, stroke or myocardial infarction without any other origin, detected by either CT scan or duplex sonography. Diagnostics were only initiated if symptoms occurred.

### Anticoagulation regimen

Patients were anticoagulated in accordance with national guidelines^14^, known independent risk factors, and pre-existing diseases, as well as body weight. The timing and dose as well as the choice of substance were decided according to the hospital’s guidelines and in interdisciplinary bedside discourse with intensive care and neurosurgical practitioners. During the course of this study, tinzaparin and nadroparin were used for prophylactic AC in standard care, unfractioned heparin (UFH) was used in intensive care. If not otherwise stated, “prophylactic AC” refers to the weight-adapted administration of heparins. The fact that AC did not follow a definite protocol resulted in the variety of timings and therapy regimen examined in this study. However, the common course of treatment and diagnosis can be described as follows:

Patients were not anticoagulated until CT or MR imaging of the head was obtained, which was routinely done on the first postoperative day (or within 24 h after surgery). CT scans were performed immediately following the onset of new neurological deficits. We began prophylactic AC treatment in patients undergoing intracranial surgery on the first or second day after surgery, as long as postoperative CT or MR imaging do not show any signs of residual bleeding. Patients with postoperative or perioperative hemorrhage are mainly anticoagulated after two to five days following a repeat CT scan without any sign of a growing hemorrhage. In patients with a preoperative regimen of direct oral anticoagulants (DOAC) or vitamin K antagonists (VKA), therapy was interrupted for up to three weeks depending on indications during the period concerned. In these cases, heparin bridging was regularly performed. AC reversal was performed for increased INR, thrombocytopenia and positive medication history for antiplatelet therapy. Tranexamic acid was administered intraoperatively in interdisciplinary consensus. During the course of this study, no standardized protocol for AC reversal was used. Since 2014, a standard operating procedure has stated that hospitalized trauma patients should be considered for AC as long as there are no contraindications, in which case either mechanical prophylaxis or no prophylaxis at all should be applied. In this study, all patients underwent surgery in an emergency setting.

### Assessed data

We assessed data of the following categories:

- Baseline data and medical history.
- Perioperative data (e.g. length of surgical procedure).
- Anticoagulation regimen pre- and postoperatively.
- Radiological data on pre- and postoperative hemorrhage.
- Thromboembolic events (entity, treatment).
- Outcome and functional status at admission.

### Statistical analysis

To describe the cohort, continuous parameters are presented as median, mean and with standard deviation; nominal parameters are displayed as percentage.

The population was dichotomously branched by the occurrence or nonoccurrence of adverse events (intracranial hemorrhage and thromboembolic event).

Continuous and ordinal parameters were evaluated with Mann-Whitney U-Test. Dichotomous parameters were analyzed using Fisher’s exact test. P-values lower than 0.05 were considered statistically significant. Odd’s Ratios and their 95% confidence interval were calculated with univariate binomial logistic regression. ROC analysis were performed for early or late intervention. All analyses were computed using IBM SPSS Statistics software version 24 (IBM, Armonk, New York State, USA).

## Results

**Fig. 2:**
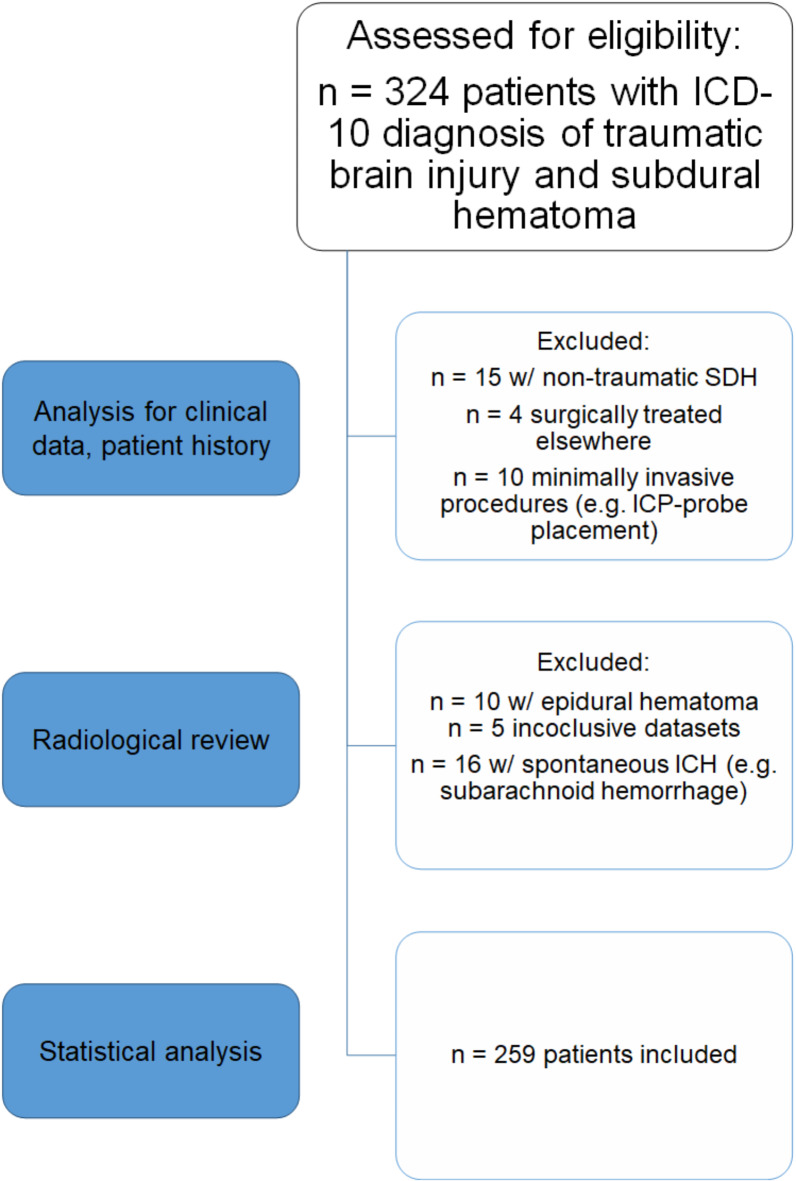
Flow chart for patient recruitment. SDH: Subdural hematoma. ICP: Intracranial pressure. ICH: intracranial hemorrhage.

*N* = 324 patients were eligible. We excluded patients with isolated extraaxial bleeding, i.e. isolated epidural hematoma (*n* = 10). Spontaneous ICB (*n* = 14) or SAH (*n* = 2) with SDH and non-traumatic (*n* = 15) SDH were excluded. *N* = 5 patients died before AC administration was possible. *N* = 5 datasets were inconclusive and *n* = 4 patients underwent surgery externally. We excluded minimally invasive procedures such as ICP-monitor placement (*n* = 10). In total, *n* = 259 patients were included in the retrospective analysis (Fig. 2).

**Table 1:** Patient characteristics. Frequencies are given in percent (%). Metric values are given as mean and standard deviation.

Patient and TBI baseline data (*n* = 259)	Frequency / Mean
Epidemiological data	
Gender (male)	175 (67.3%)
Age (years)	69.3 (± 16.4)
BMI (kg/m²)	25.1 (± 4.1)
Pre-operative therapeutic AC	73 (28.2%)
- Anti-platelet therapy	52 (20.1%)
Coagulopathy	11 (4.2%)
Atrial fibrillation	64 (24.7%)
Hypertension	159 (61.4%)
Diabetes	60 (23.2%)
Elevated blood-alcohol	39 (15.1%)
High-speed trauma	47 (18.1%)
Radiological assessment	
Isolated acute subdural hematoma	213 (82.2%)
- Suspected pre-existing chronic subdural hematoma	51 (19.7%)
- Additional intracerebral hemorrhage (contusion)	63 (24.3%)
- Additional epidural hematoma	8 (3.1%)
- Additional traumatic subarachnoid hemorrhage	64 (24.7%)
Maximum width of SDH (mm)	15.2 (± 7.29)
Midline shift (mm)	8.95 (± 6.92)
Radiological signs of Herniation	34 (13.1%)
Basic neurological assessment	
Mild TBI	106 (40.9%)
Moderate TBI	31 (12.0%)
Severe TBI	122 (47.1%)
GCS at admission	9.1 (± 5.1)
Unilateral Anisocoria	34 (13.1%)
Bilateral Anisocoria	33 (12.7%)
Under CPR	5 (1.9%)
Course of treatment	
Time until surgery (h)	1.6 ± 9.3
Time from first surgery until PH (h)	8.96 ± 8.0
Duration of surgery (min)	92.4 ± 39.5
Complication	
Thromboembolic event	13 (5.0%)
DVT	4 (1.5%)
Systemic (e.g. mesenterial)	4 (1.5%)
- Cerebral ischemia (not related to surgery)	4 (1.5%)
- Myocardial infarction	1 (0.3%)
PH within 30 days	34 (13.1%)
- With surgical revision	28 (11.2%)
Death within 30 days	65 (25.1%)
Heparin regimen	
Prophylactic AC (within ICU treatment)	212 (81.9%)
UFH	47 (18.1%)
- LMWH	165 (63.5%)
Heparin within 24 h or less	9 (3.5%)
Heparin within 48 h or less	151 (58.1%)
Therapeutic AC	1 (0.3%)
Time point of initiation of heparin in h	74.3 (± 54.1)

AC: Anticoagulation. BMI: Body Mass Index. GCS: Glasgow Coma Scale. SDH: Subdural Hematoma. CPR: Cardiopulmonary Resuscitation. DVT: Deep vein thrombosis. UFH: unfractioned heparin. LMWH: low molecular-weight heparin. PH: Postoperative hemorrhage.

### Patient characteristics

Baseline data is presented in Table 1. Patients were mostly male (67.3%) and mean age was 69.3 (± 16.4). Preexisting therapeutic AC was rather common (*n* = 73, 28.2%). Comorbidities were age-typical). Classic acute subdural hematoma was most common (*n* = 213, 82.2%), while 51 (19.7%) patients showed signs of preexisting chronic subdural hematoma as underlying disease. Additional, trauma-related intracranial hemorrhage (ICH) occurred in 63 (24.3%) patients. Mean Glasgow Coma Scale (GCS) value was 9.1 (± 5.1), while most patients suffered from severe TBI (*n* = 122, 47.1%), followed by mild TBI (*n* = 106, 40.9%) and moderate TBI (*n* = 31, 12.0%). 65 patients (25.1%) died within 30 days of follow-up. 1 patient was treated with therapeutic AC, in this case antiplatelet therapy due to myocardial infarction.

Average time to surgery (initial diagnostic until skin incision) was 1.6 h, including transfer time from external facilities, while time of surgery was averagely 92.4 (± 39.5) minutes.

TE occurred in 14 cases (5.4%), with *n* = 6 cases of cerebral thromboembolic ischemia (2.3%) *n* = 3 cases of deep vein thrombosis (1.2%), *n* = 4 cases of systemic embolism such as mesenterial infarction (1.5%) and *n* = 1 myocardial infarction (0.3%). PH occurred in 34 (13.1%) patients, whereas 28 patients (10.8%) had to be revised surgically. Most patients were given prophylactic AC within the course of post-operative treatment (*n* = 212, 81.9%) according to the regimen stated above. The minority of patients was anticoagulated early on (< 24 h: *n* = 9, 3.5%), while most patients were anticoagulated according to standard of care within 48 h (*n* = 151, 58.1%). Due to the aforementioned delay of AC initiation, based on individual therapy decisions, average time of AC administration was 74.3 h, or roughly 3 days.

**Table 2:** Risk factors for postoperative hemorrhage and thromboembolic events in patients suffering from traumatic brain injury and surgical evacuation of subdural hematoma.

	Thromboembolic events			Postoperative hemorrhage		
	w/o(*n* = 245)	with (*n* = 14)	p-value	w/o (*n* = 224)	with (*n* = 34)	p-value
Age	69.76 ± 16.22	61.71 ± 19.12	0.145	68.88 ± 16.86	72.76 ± 12.92	0.207
Sex (male)	163	12	0.327	150	25	0.683
BMI	25.7 ± 4.1	25.0 ± 3.53	0.456	25.65 ± 3.94	26.03 ± 4.84	0.669
Preoperative therapeutic AC Anti-platelet therapy	71 49	2 3	0.469 0.342	63 42	10 10	0.036* 0.270
Hypertension	150	9	0.529	137	22	0.422
Coagulopathy	9	2	0.057	8	3	0.167
Atrial fibrillation	63	1	0.117	53	11	0.188
Midline-shift	8.69 ± 6.72	13.27 ± 8.82	0.119	8.82 ± 6.99	9.66 ± 6.57	0.525
Maximum width of SDH	15.2 ± 7.2	14.7 ± 7.7	0.837	15.02 ± 7.38	15.95 ± 6.79	0.543
Herniation	29	5	0.078	27	7	0.033*
Dilated pupil	30	4	0.109	28	6	0.006*
Death within 30 days	60	5	0.357	51	14	0.022*
Hypertension	150	9	0.819	137	22	0.422
Elevated blood alcohol level	36	3	0.512	36	3	0.192
Time until surgery (h)	2.82 ± 9.63	1.86 ± 0.26	0.01*	2.91 ± 9.97	1.15 ± 3.6	0.057
Duration of surgery (min)	92.75 ± 39.7	86.36 ± 36.5	0.536	90.67 ± 39.4	104.6 ± 39.6	0.067
Heparin within 24 h or less	9	0	n.a.	9	0	n.a.
Heparin within 48 h or less	140	11	0.186	148	3	0.006*
Time point of initiation of heparin in h	73.61 ± 55.26	84 ± 27.3	0.211	68.78 ± 32.8	113.56 ± 122.57	0.010*

BMI: Body Mass Index. AC: Anticoagulation. SDH: Subdural hematoma.

### Prophylactic anticoagulation and risk for postoperative intracranial hemorrhage

Patients suffering from PH were more likely to die within 30 days (*p* = 0.022, OR = 2,361 CI 1.114–5.003), making recurrent intracranial hemorrhage the most relevant outcome-relevant event in course of treatment. A significant risk factor for PH were pre-existing therapeutic AC (*p* = 0.036), whereas anti-platelet therapy was not correlated with PH (*p* = 0.270). Preoperative herniation with the clinical aspect of a dilated pupil was significantly associated with PH (*p* = 0.006), while midline shift (*p* = 0.525) and maximum width of the subdural hematoma (*p* = 0.543) were not. Patients who suffered from PH were given prophylactic AC significantly later (> 48 h: *p* = 0.010, time-dependent: *p* = 0.010, s. Table 2).

### Prophylactic anticoagulation and thromboembolic events

With the rare occurrence of TE, we did not detect any significant risk factors or correlations. Early (< 24 h) or timely (< 48 h) prophylactic AC did not decrease the occurrence of symptomatic TE significantly. Patients being operated on with less delay were more likely to suffer from TE (*p* = 0.010).

### Timing of prophylactic AC

**Fig. 3:**
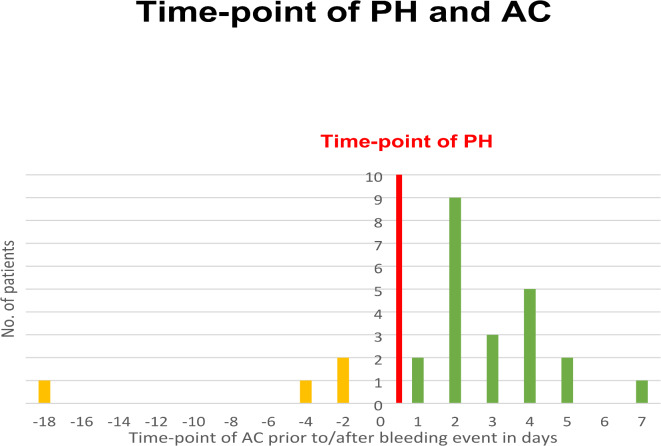
The timing of prophylactic anticoagulation (AC) in time-dependency to PH event. 4 patients were administered AC before the event (18,4 and 2 days (*n* = 2) before PH occurred), while the majority of 30 patients with PH was administered AC later on.

When connecting the time of AC administration and PH events, the missing causality between the two becomes clearer: Of 34 patients suffering from PH, only 4 were anticoagulated before the PH event (Fig. 3), while the majority of 30 patients was administered AC after the event itself. The average time from wound closure until PH was ~ 9 h.

## Discussion

### Limitations

Obviously, design and power are not sufficient to prove efficacy or safety of early AC administration. Additionally, the study is limited by the rare occurrence of adverse events in general, especially TE, making conclusions in that regard difficult. Moreover, in the retrospective analysis, no conclusion can be drawn about the causality of the relationships.

### Early anticoagulation and risk factors for recurrent hemorrhage

We found no increase in occurrence of PH due to (early) application of prophylactic AC, which aligns with preliminary results^15,16^. By putting AC administration and bleeding events on the timeline, we were able to clarify the association between medication and complication, making a causality highly unlikely. In our cohort, only 4 patients suffering from PH were exposed to AC. Any statistical evaluation of this extreme scarcity of cases is in vain, while there is a significant risk for selection bias.

The negative effect of recurrent bleeding on overall survival and outcome remains undisputed by the results. The preoperative status of therapeutic AC and the clinical state remain most influential on the occurrence of PH.

Radiological signs for herniation and a dilated pupil were correlated to increased PH rates, while the sheer size of the hematoma and the resulting midline shift itself were not. This might support the thesis of acute hemorrhage into pre-formed intracranial spaces, such as chronic hematoma. Another explanation is the atrophy in this age group, allowing for a rather severe brain shift without putting vital structures at risk. The sign of an impaired oculomotor function keeps it utmost importance in clinical evaluation. While GCS at admission had no influence on PH rates (*p* = 0.356), its predictive value for overall mortality remains highly significant (*p* < 0.001).

Alcohol consumption seems to be a common co-incidence in traumatic brain injury. This might come as no surprise, yet it underlines the danger of abuse beyond the sequelae of chronic exposure.

### Prophylactic anticoagulation as a mean to prevent thromboembolic events

In the clinical routine that was maintained during the time this retrospective covers, there were no regular screenings for DVT, pulmonary embolism or other TE. TE were only documented, if clinical signs occurred, i.e. they became symptomatic. Treatment was then initiated based on applicable guidelines. On this basis, the detection of TE in this cohort might be falsely low. Although the absence of pulmonary embolism in the examined collective is pleasant, it is highly unusual.

While the efficacy and safety of heparin in the prevention of TE has been well investigated, the time-point of administration of AC in the perioperative routine should be considered in future studies. In contrast to PH and with the absence of pulmonary embolism, TE did not increase mortality in this cohort.

We found a significant correlation regarding time between CT scan and surgery and the development of TE (*p* = 0.010). Counterintuitively, a shorter time-span on the way to the OR contributed to a higher rate of TE. While an easy explanation is hard to find, one could theorize, that more severe cases with worse clinical presentation were transferred to the OR faster; in the long-run, these patients are more prone to TE. In contrast, a shorter CT-to-OR time did NOT result in higher rates of PH, which were significantly correlated to more severe cases.

### Limitations

The study is limited per design to draw conclusion on causality. The cohort is most heterogeneous in injury severity. Concomitant injuries are most influential on both physiological coagulation and clinical decision making on when to initiate prophylactic AC. Other surgeries than cranial have not been taken into account.

## Conclusion

We found no evidence of a contribution of timely prophylactic AC to PH in patients suffering from traumatic subdural hematoma. While its efficacy and safety in prevention of TE has been investigated elsewhere, early application did not increase the risk for postoperative PH in this cohort.

Initial clinical status, as displayed by radiological and clinical signs of herniation, might be rather determining for PH in subdural hematoma than prophylactic anticoagulation. Pre-operative anticoagulation medication showed to be influential. PH itself increases overall mortality.

## Data Availability

The datasets used and/or analyzed during the current study are available from the corresponding author on reasonable request.
